# Zn^2+^ Inhibits Coronavirus and Arterivirus RNA Polymerase Activity *In Vitro* and Zinc Ionophores Block the Replication of These Viruses in Cell Culture

**DOI:** 10.1371/journal.ppat.1001176

**Published:** 2010-11-04

**Authors:** Aartjan J. W. te Velthuis, Sjoerd H. E. van den Worm, Amy C. Sims, Ralph S. Baric, Eric J. Snijder, Martijn J. van Hemert

**Affiliations:** 1 Molecular Virology Laboratory, Department of Medical Microbiology, Center of Infectious Diseases, Leiden University Medical Center, Leiden, The Netherlands; 2 Departments of Epidemiology and Microbiology and Immunology, University of North Carolina at Chapel Hill, Chapel Hill, North Carolina, United States of America; University of California San Francisco, United States of America

## Abstract

Increasing the intracellular Zn^2+^ concentration with zinc-ionophores like pyrithione (PT) can efficiently impair the replication of a variety of RNA viruses, including poliovirus and influenza virus. For some viruses this effect has been attributed to interference with viral polyprotein processing. In this study we demonstrate that the combination of Zn^2+^ and PT at low concentrations (2 µM Zn^2+^ and 2 µM PT) inhibits the replication of SARS-coronavirus (SARS-CoV) and equine arteritis virus (EAV) in cell culture. The RNA synthesis of these two distantly related nidoviruses is catalyzed by an RNA-dependent RNA polymerase (RdRp), which is the core enzyme of their multiprotein replication and transcription complex (RTC). Using an activity assay for RTCs isolated from cells infected with SARS-CoV or EAV—thus eliminating the need for PT to transport Zn^2+^ across the plasma membrane—we show that Zn^2+^ efficiently inhibits the RNA-synthesizing activity of the RTCs of both viruses. Enzymatic studies using recombinant RdRps (SARS-CoV nsp12 and EAV nsp9) purified from *E. coli* subsequently revealed that Zn^2+^ directly inhibited the *in vitro* activity of both nidovirus polymerases. More specifically, Zn^2+^ was found to block the initiation step of EAV RNA synthesis, whereas in the case of the SARS-CoV RdRp elongation was inhibited and template binding reduced. By chelating Zn^2+^ with MgEDTA, the inhibitory effect of the divalent cation could be reversed, which provides a novel experimental tool for *in vitro* studies of the molecular details of nidovirus replication and transcription.

## Author Summary

Positive-stranded RNA (+RNA) viruses include many important pathogens. They have evolved a variety of replication strategies, but are unified in the fact that an RNA-dependent RNA polymerase (RdRp) functions as the core enzyme of their RNA-synthesizing machinery. The RdRp is commonly embedded in a membrane-associated replication complex that is assembled from viral RNA, and viral and host proteins. Given their crucial function in the viral replicative cycle, RdRps are key targets for antiviral research. Increased intracellular Zn^2+^ concentrations are known to efficiently impair replication of a number of RNA viruses, e.g. by interfering with correct proteolytic processing of viral polyproteins. Here, we not only show that corona- and arterivirus replication can be inhibited by increased Zn^2+^ levels, but also use both isolated replication complexes and purified recombinant RdRps to demonstrate that this effect may be based on direct inhibition of nidovirus RdRps. The combination of protocols described here will be valuable for future studies into the function of nidoviral enzyme complexes.

## Introduction

Zinc ions are involved in many different cellular processes and have proven crucial for the proper folding and activity of various cellular enzymes and transcription factors. Zn^2+^ is probably an important cofactor for numerous viral proteins as well. Nevertheless, the intracellular concentration of free Zn^2+^ is maintained at a relatively low level by metallothioneins, likely due to the fact that Zn^2+^ can serve as intracellular second messenger and may trigger apoptosis or a decrease in protein synthesis at elevated concentrations [1], [2], [3]. Interestingly, in cell culture studies, high Zn^2+^ concentrations and the addition of compounds that stimulate cellular import of Zn^2+^, such as hinokitol (HK), pyrrolidine dithiocarbamate (PDTC) and pyrithione (PT), were found to inhibit the replication of various RNA viruses, including influenza virus [4], respiratory syncytial virus [5] and several picornaviruses [6], [7], [8], [9], [10], [11]. Although these previous studies provided limited mechanistic information, this suggests that intracellular Zn^2+^ levels affect a common step in the replicative cycle of these viruses.

In cell culture, PT stimulates Zn^2+^ uptake within minutes and inhibits RNA virus replication through a mechanism that has only been studied in reasonable detail for picornaviruses [11], [12]. *In vitro* studies with purified rhinovirus and poliovirus 3C proteases revealed that protease activity was inhibited by Zn^2+^ [13], [14], which is in line with the inhibition of polyprotein processing by zinc ions that was observed in cells infected with human rhinovirus and coxsackievirus B3 [11]. The replication of segmented negative-strand RNA viruses such as influenza virus, however, does not depend on polyprotein processing and the effect of PDTC-mediated Zn^2+^ import was therefore hypothesized to result from inhibition of the viral RNA-dependent RNA polymerase (RdRp) and cellular cofactors [4]. Moreover, an inhibitory effect of Zn^2+^ on the activity of purified RdRps from rhinoviruses and hepatitis C virus was noted, but not investigated in any detail [15], [16].

Details on the effect of zinc ions are currently largely unknown for nidoviruses. This large group of positive-strand RNA (+RNA) viruses includes major pathogens of humans and livestock, such as severe acute respiratory syndrome coronavirus (SARS-CoV), other human coronaviruses, the arteriviruses equine arteritis virus (EAV), and porcine reproductive and respiratory syndrome virus (PRRSV) [17], [18]. The common ancestry of nidoviruses is reflected in their similar genome organization and expression strategy, and in the conservation of a number of key enzymatic functions in their large replicase polyproteins [19]. A hallmark of the corona- and arterivirus replicative cycle is the transcription of a 5′- and 3′-coterminal nested set of subgenomic (sg) mRNAs from which the viral structural and accessory protein genes are expressed [20], [21].

Analogous to picornaviruses [13], [22], zinc ions were demonstrated to inhibit certain proteolytic cleavages in the processing of the coronavirus replicase polyproteins in infected cells and cell-free systems [23], [24]. In this study we report that the zinc-ionophore pyrithione (PT) in combination with Zn^2+^ is a potent inhibitor of the replication of SARS-coronavirus (SARS-CoV) and equine arteritis virus (EAV) in cell culture. To assess whether - besides a possible effect on proteolytic processing - nidovirus RTC subunits and RNA synthesis are directly affected by Zn^2+^, we employed *in vitro* systems for SARS-CoV and EAV RNA synthesis that are based on membrane-associated RTCs isolated from infected cells (from here on referred to as RTC assays) [25], [26]. In addition, we used *in vitro* recombinant RdRp assays to directly study the effect of zinc ions on the RdRps of SARS-CoV and EAV [27], [28].

Using these independent *in vitro* approaches, we were able to demonstrate that Zn^2+^ directly impairs nidovirus RNA synthesis, since it had a strong inhibitory effect in both RTC and RdRp assays. Interestingly, the Zn^2+^-mediated inhibition could be reversed through the addition of a Zn^2+^ chelator (MgEDTA). We therefore applied this compound to stop and restart the *in vitro* RNA-synthesizing activity at will. This convenient tool allowed us to study various mechanistic aspects of arteri- and coronavirus RNA synthesis in more detail. Additionally, the zinc-mediated inhibition of nidovirus RNA synthesis described here may provide an interesting basis to further explore the use of zinc-ionophores in antiviral therapy.

## Results

### Zinc and pyrithione inhibit nidovirus replication *in vivo*

Zinc ions are involved in many different cellular processes, but the concentration of free Zn^2+^ is maintained at a relatively low level by metallothioneins [1]. Zn^2+^ and compounds that stimulate import of Zn^2+^ into cells, such as PT, were previously found to inhibit replication of several picornaviruses, including rhinoviruses, foot-and-mouth disease virus, coxsackievirus, and mengovirus in cell culture [6], [7], [8], [9], [10], [11]. To determine whether Zn^2+^ has a similar effect on nidoviruses, we investigated the effect of PT and Zn^2+^ on the replication of EAV and SARS-CoV in Vero-E6 cells, using reporter viruses that express green fluorescent proteins (GFP), i.e., EAV-GFP [29] and SARS-CoV-GFP [30]. EAV-GFP encodes an N-terminal fusion of GFP to the viral nonstructural protein 2 (nsp2), one of the cleavage products of the replicase polyproteins, and thus provides a direct readout for translation of the replicase gene. In SARS-CoV-GFP, reporter expression occurs from sg mRNA 7, following the replacement of two accessory protein-coding genes (ORFs 7a and 7b) that are dispensable for replication in cell culture.

**Figure 1:**
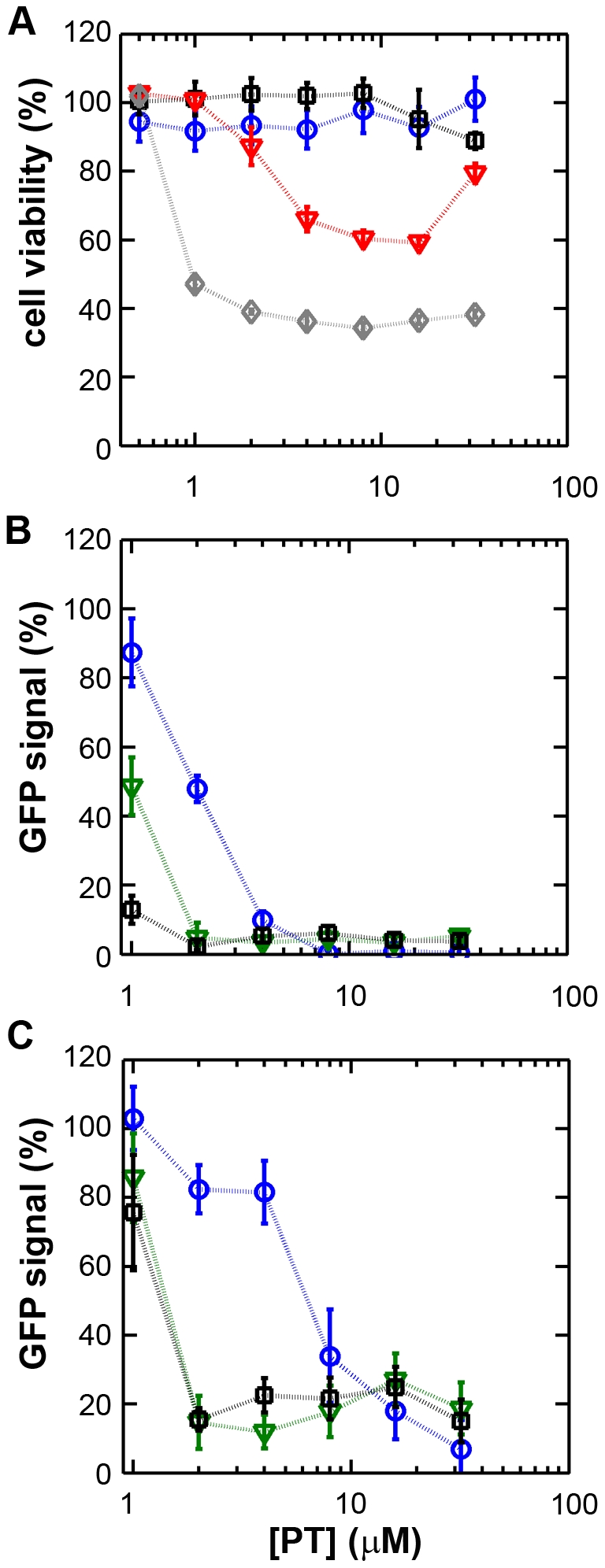
The zinc ionophore pyrithione inhibits nidovirus replication in cell culture. (**A**) Cytotoxicity of PT in Vero-E6 cells in the absence (blue circles) or presence of 2 (black squares), 4 (red triangles), or 8 µM (gray diamonds) ZnOAc_2_ as determined by the MTS assay after 18 hours of exposure. (**B**) Dose-response curves showing the effect of PT and Zn^2+^ on the GFP fluorescence in Vero-E6 cells infected with a GFP-expressing EAV reporter strain at 17 h p.i. Data were normalized to GFP expression in infected, untreated control cultures (100%). The different Zn^2+^ concentrations added to the medium were 0 (blue circles), 1 (green triangles), or 2 µM ZnOAc_2_ (black squares). (**C**) Effect of PT and Zn^2+^ on the GFP fluorescence in Vero-E6 cells infected with a GFP-expressing SARS-CoV reporter strain at 17 h p.i. Data were normalized to GFP expression in infected untreated control cells (100%). Colors for different Zn^2+^ concentrations as in Fig. 1B. Error bars indicate the standard deviation (n = 4).

We first assessed the cytotoxicity of a range of PT concentrations (0–32 µM) in the presence of 0 to 8 µM ZnOAc_2_. Treatment with PT of concentrations up to 32 µM in combination with <4 µM ZnOAc_2_ did not reduce the viability of mock-infected cells after 18 h (Fig. 1A), as measured by the colorimetric MTS (3-(4,5-dimethylthiazol-2-yl)-5-(3-carboxymethoxyphenyl)-2-(4-sulfophenyl)-2H-tetrazolium) viability assay. As elevated Zn^2+^ concentrations are known to inhibit cellular translation, we also used metabolic labeling with ^35^S-methionine to assess the effect of PT and Zn^2+^ on cellular protein synthesis. Incubation of Vero-E6 cells for 18 h with the combinations of PT and Zn^2+^ mentioned above, followed by a 2-h metabolic labeling, revealed no change in overall cellular protein synthesis when the concentration of ZnOAc_2_ was <4 µM (data not shown).

Using these non-cytotoxic conditions we subsequently tested the effect of PT and ZnOAc_2_ on EAV-GFP and SARS-CoV-GFP replication. To this end, Vero-E6 cells in 96-well plates were infected with a multiplicity of infection (m.o.i.) of 4. One hour post infection (h p.i.), between 0 and 32 µM of PT and 0, 1, or 2 µM ZnOAc_2_ were added to the culture medium. At 17 h p.i., a time point at which GFP expression in untreated infected cells reaches its maximum for both viruses, cells were fixed, and GFP fluorescence was quantified.

The reporter gene expression of both SARS-CoV-GFP and EAV-GFP was already significantly inhibited in a dose-dependent manner by the addition of PT alone (Fig. 1B and C). This effect was significantly enhanced when 2 µM of Zn^2+^ was added to the medium. We found that addition of ZnOAc_2_ alone also reduced virus replication, but only at levels that were close to the 50% cytotoxicity concentration (CC_50_) of ZnOAc_2_ in Vero-E6 cells (∼70 µM, data not shown). This is likely due to the poor solubility of Zn^2+^ in phosphate-containing medium and the inefficient uptake of Zn^2+^ by cells in the absence of zinc-ionophores. The combination of 2 µM PT and 2 µM ZnOAc_2_ resulted in a 98±1% and 85±3% reduction of the GFP signal for EAV-GFP and SARS-CoV-GFP, respectively. No cytotoxicity was observed for this combination of PT and ZnOAc_2_ concentrations. From the dose-response curves in Fig. 1, a CC_50_ value of 82 µM was calculated for PT in the presence of 2 µM zinc. Half maximal inhibitory concentrations (IC_50_) of 1.4 µM and 0.5 µM and selectivity indices of 59 and 164 were calculated for SARS-CoV and EAV, respectively.

### Zn^2+^ reversibly inhibits the RNA-synthesizing activity of isolated nidovirus RTCs

**Figure 2:**
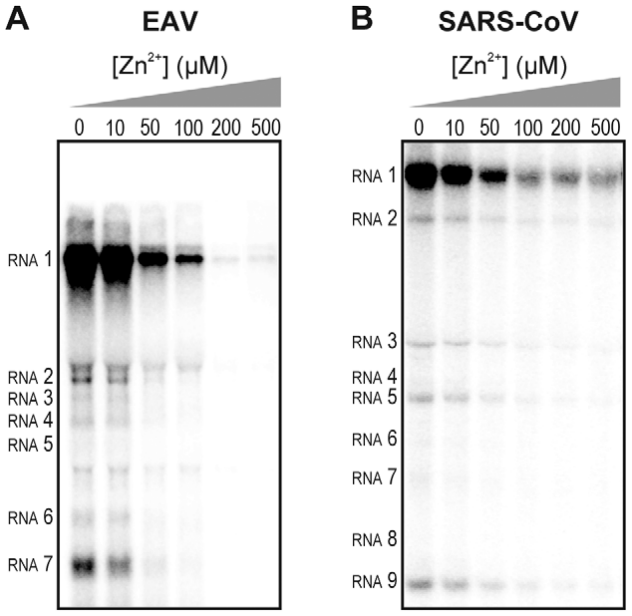
Inhibition of the *in vitro* RNA-synthesizing activity of isolated RTCs by Zn^2+^. Incorporation of [α-^32^P]CMP into viral RNA by EAV (**A**) and SARS-CoV (**B**) in RTC assays in the presence of various Zn^2+^ concentrations, as indicated above each lane.

We previously developed assays to study the *in vitro* RNA-synthesizing activity of RTCs isolated from cells infected with SARS-CoV or EAV [25], [26]. In these RTC assays [α-^32^P]CMP is incorporated into both genomic (replication) and sg mRNA (transcription) (Fig. 2). This allowed us to monitor the synthesis of the same viral RNA molecules that can be detected by hybridization of RNA from nidovirus-infected cells. A benefit of these assays is that the activity does not depend on continued protein synthesis and that it allows us to study viral RNA synthesis independent of other aspects of the viral replicative cycle [26]. To investigate whether the inhibitory effect of PT and zinc ions on nidovirus replication in cell culture is reflected in a direct effect of Zn^2+^ on viral RNA synthesis, we tested the effect of Zn^2+^ addition on RTC activity. For both EAV (Fig. 2A) and SARS-CoV (Fig. 2B), a dose-dependent decrease in the amount of RNA synthesized was observed when ZnOAc_2_ was present. For both viruses, a more than 50% reduction of overall RNA-synthesis was observed at a Zn^2+^ concentration of 50 µM, while less than 5% activity remained at a Zn^2+^ concentration of 500 µM. Both genome synthesis and sg mRNA production were equally affected.

**Figure 3:**
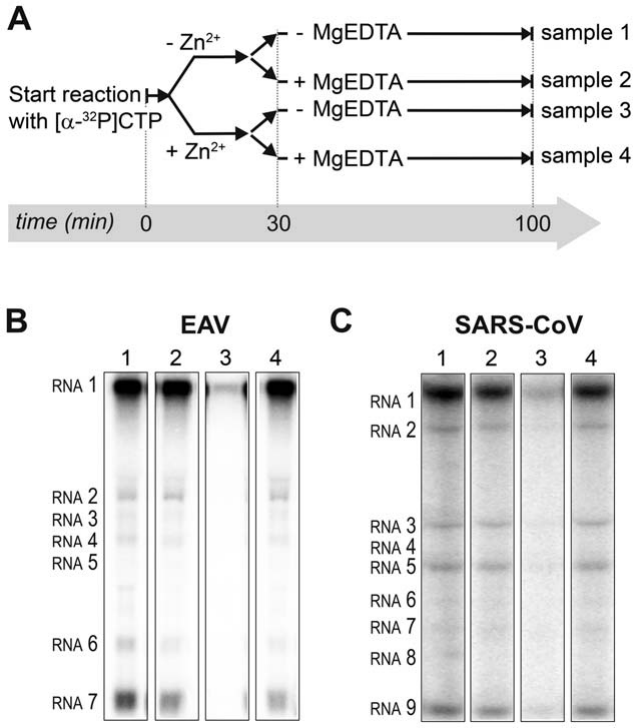
Inhibition of nidovirus RTC activity by Zn^2+^ can be reversed by chelation. (**A**) Schematic representation of the *in vitro* assays with isolated RTCs, which were initiated with [α-^32^P]CTP, either in the absence (sample 1 and 2) or presence of 500 µM Zn^2+^. After a 30-min incubation at 30°C, both the untreated and Zn^2+^-treated samples were split into two aliquots, and 1 mM of the Zn^2+^ chelator MgEDTA was added to samples 2 and 4. All reactions were subsequently incubated for another 70 min before termination. (**B**) Analysis of RNA products synthesized in assays with EAV RTCs. Numbers above the lanes refer to the sample numbers described under (A). (**C**) *In vitro* activity assay with SARS-CoV RTCs.

To test whether the inhibition of RTC activity by Zn^2+^ was reversible, RTC reactions were started in the presence or absence of 500 µM Zn^2+^. After 30 min, these reactions were split into two aliquots and magnesium-saturated EDTA (MgEDTA) was added to one of the tubes to a final concentration of 1 mM (Fig. 3A). We used MgEDTA as Zn^2+^ chelator in these *in vitro* assays, because it specifically chelates Zn^2+^ while releasing Mg^2+^, due to the higher stability constant of the ZnEDTA complex. Uncomplexed EDTA inhibited RTC activity in all reactions (data not shown), most likely by chelating the Mg^2+^ that is crucial for RdRp activity [27], [28], whereas MgEDTA had no effects on control reactions without Zn^2+^ (Fig. 3B, compare lane 1 and 2). As shown in Fig. 2, the EAV RTC activity that was inhibited by Zn^2+^ (Fig. 3B&C, lane 3) could be restored by the addition of MgEDTA (Fig. 3B, lane 4) to a level observed for control reactions without Zn^2+^ (Fig. 3B, lane 1). Compared to the untreated control, the EAV RTC assay produced approximately 30% less RNA, which was consistent with the 30% shorter reaction time after the addition of the MgEDTA (100 versus 70 min for lanes 1 and 4, respectively). Surprisingly, SARS-CoV RTC assays that were consecutively supplemented with Zn^2+^ and MgEDTA incorporated slightly more [α-^32^P]CMP compared to untreated control reactions (Fig. 3C; compare lane 1 and 4). This effect was not due to chelation of the Zn^2+^ already present in the post-nuclear supernatant (PNS) of SARS-CoV-infected cells, as this increase was not observed when MgEDTA was added to a control reaction without additional Zn^2+^ (Fig. 3C, lane 2).

### Zinc ions affect the in vitro activity of recombinant nidovirus RdRps

**Figure 4:**
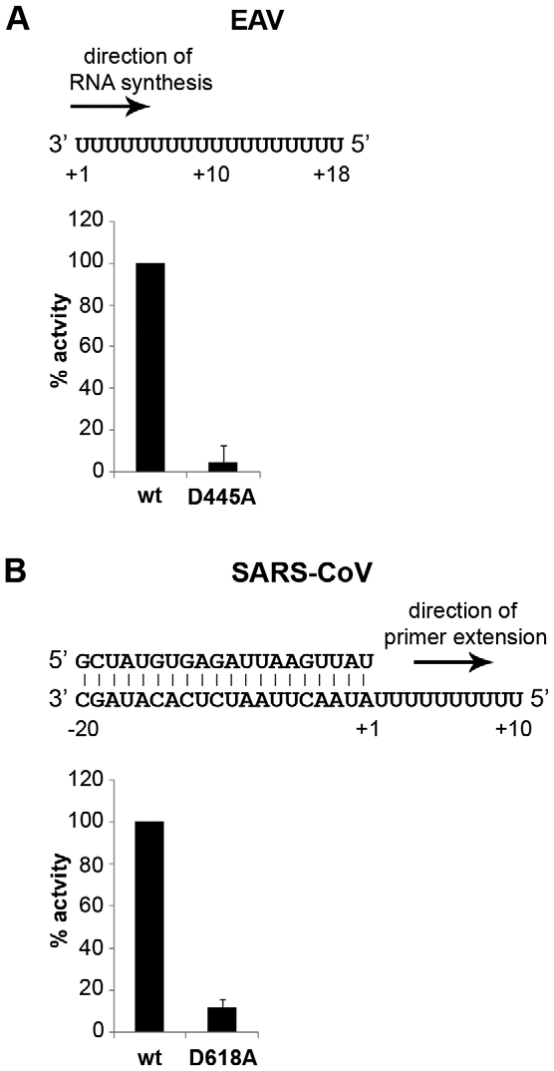
EAV and SARS-CoV RdRp assays with wild-type enzyme and active-site mutants. (**A**) The EAV polymerase was incapable of primer extension and required a free 3′ end and poly(U) residues to initiate. Nucleotide incorporating activity of the wild-type enzyme and D445A mutant of nsp9 on an 18-mer poly(U) template confirmed the specificity of our assay. (**B**) SARS-CoV nsp12 RdRp assays were performed with an RNA duplex with a 5′ U_10_ overhang as template. The bar graph shows the nucleotide incorporating activities of wild-type and D618A nsp12. Error bars represent standard error of the mean (n = 3).

To establish whether inhibition of RTC activity might be due to a direct effect of Zn^2+^ on nidovirus RdRp activity, we tested the effect of Zn^2+^ on the activity of the purified recombinant RdRps of SARS-CoV (nsp12) and EAV (nsp9) using previously developed RdRp assays [27], [28]. Using an 18-mer polyU template, the EAV RdRp incorporated [α-^32^P]AMP into RNA products of up to 18 nt in length (Fig. 4A). Initiation was *de novo*, which is in line with previous observations and the presence of a conserved priming loop in the nsp9 sequence [28]. Unlike the EAV RdRp nsp9, the *in vitro* activity of the SARS-CoV RdRp nsp12 - which lacks a priming loop - was shown to be strictly primer-dependent [27]. Thus, to study the RdRp activity of SARS-CoV nsp12, a primed polyU template was used (Fig. 4B), thereby allowing us to sample [α-^32^P]AMP incorporation as described previously [27]. As specificity controls, we used the previously described SARS-CoV nsp12 mutant D618A [27], which contains an aspartate to alanine substitution in motif A of the RdRp active site, and EAV nsp9-D445A, in which we engineered an aspartate to alanine substitution at the corresponding site of EAV nsp9 [28], [31]. Both mutant RdRps showed greatly reduced [α-^32^P]AMP incorporation in our assays (Fig. 4), confirming once again that the radiolabeled RNA products derived from nidovirus RdRp activity.

**Figure 5:**
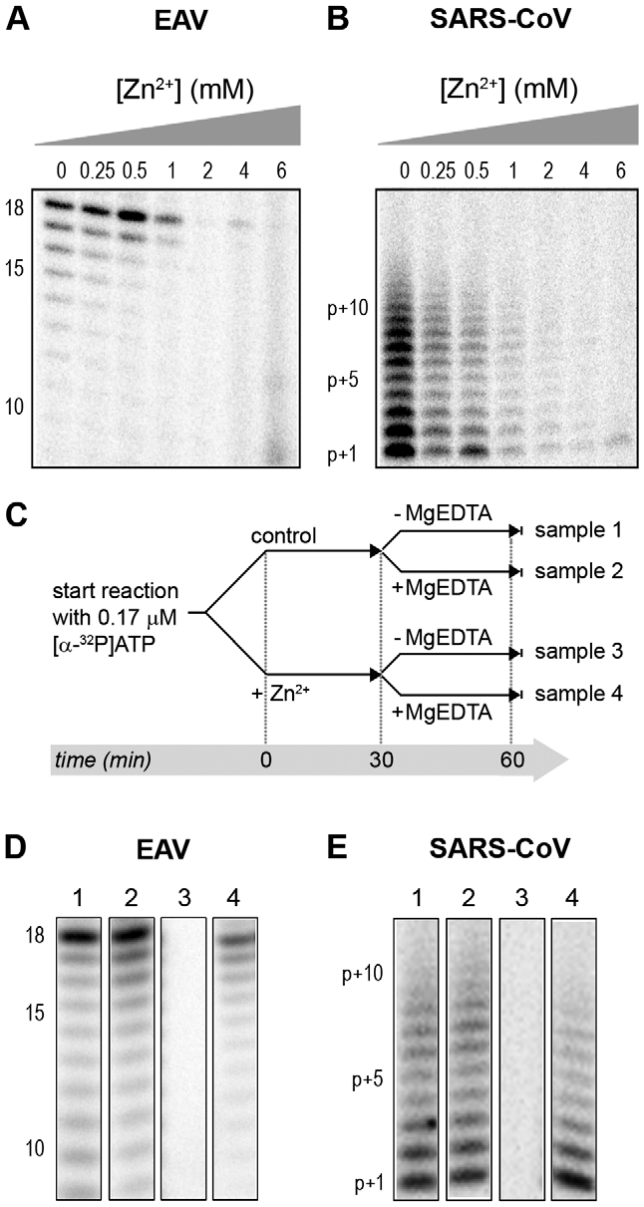
The activity of the RdRps of EAV and SARS-CoV is reversibly inhibited by Zn^2+^. RdRp activity of purified EAV nsp9 (**A**) and SARS-CoV nsp12 (**B**) in the presence of various Zn^2+^ concentrations, as indicated above the lanes. (**C**) Schematic representation of the experiment to test whether Zn^2+^-mediated inhibition of RdRp activity could be reversed with MgEDTA. RdRp reactions, either untreated controls (sample 1 and 2) or reactions containing 6 mM Zn^2+^ (samples 3 and 4) were incubated for 30 min. Both Zn^2+^-containing and control samples were split into two aliquots and 6 mM MgEDTA was added to sample 2 and 4. All reactions were incubated for an additional 30 min and then terminated. Reaction products of the RdRp assays with EAV nsp9 and SARS-CoV nsp12 are shown in (**D**) and (**E**), respectively. Numbers above the lanes refer to the sample numbers described under (C).

Addition of ZnOAc_2_ to RdRp assays resulted in a strong, dose-dependent inhibition of enzymatic activity for both the EAV and SARS-CoV enzyme (Fig. 5A and B, respectively), similar to what was observed in RTC assays. In fact, compared to other divalent metal ions such Co^2+^ and Ca^2+^, which typically bind to amino acid side chains containing oxygen atoms rather than sulfur groups, Zn^2+^ was the most efficient inhibitor of SARS-CoV nsp12 RdRp activity (Supplemental Fig. S1). To test whether, as in the RTC assay, the RdRp inhibition by zinc ions was reversible, RdRp assays were pre-incubated with 6 mM Zn^2+^, a concentration that consistently gave >95% inhibition. After 30 min, 8 mM MgEDTA was added to both a control reaction and the reaction inhibited with ZnOAc_2_, and samples were incubated for another 30 min (Fig. 5C). As shown in Fig. 5D, the inhibition of EAV RdRp activity by Zn^2+^ could be reversed by chelation of Zn^2+^ (Fig. 5D; compare lanes 3 and 4). The amount of product synthesized was consistently 60±5% of that synthesized in a 60-min control reaction (Fig. 5D; compare lanes 1 and 4), which was within the expected range given the shorter reaction time. The inhibition of the SARS-CoV RdRp was reversible as well. During the 30-min incubation after the addition of MgEDTA, SARS-CoV nsp12 incorporated 40±5% of the label incorporated during a standard 60-min reaction (Fig. 5E). This was slightly lower than the expected yield and may be caused by the elevated Mg^2+^ concentration, which was shown to be suboptimal for nsp12 activity [27] and results from the release of Mg^2+^ from MgEDTA upon chelation of Zn^2+^.

### Differential effect of Zn^2+^ on the initiation and elongation phase of nidovirus RNA synthesis

**Figure 6:**
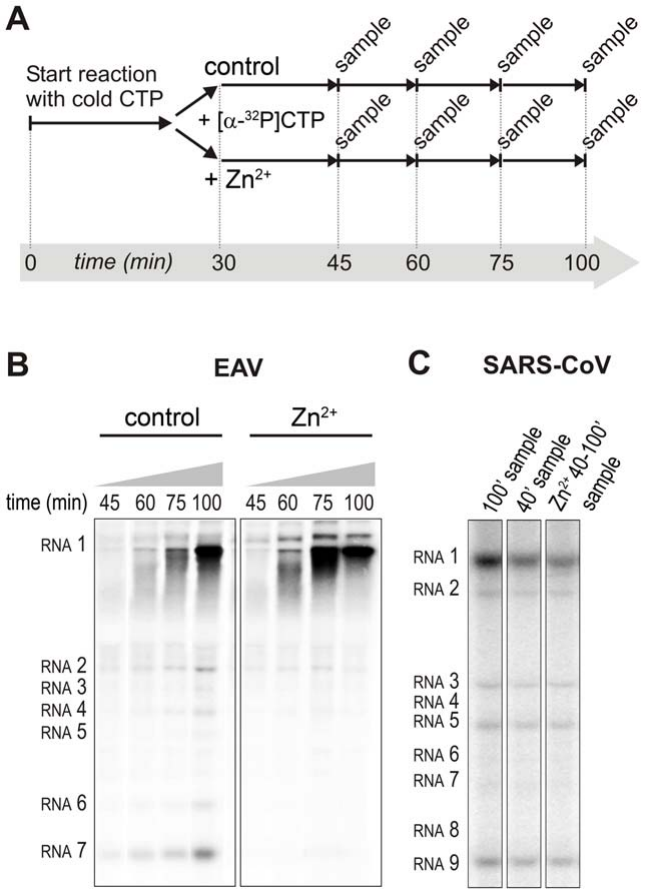
Effect of Zn^2+^ on initiation and elongation in *in vitro* assays with isolated EAV and SARS-CoV RTCs. (**A**) An *in vitro* RTC assay with isolated EAV RTCs was allowed to initiate with unlabeled NTPs (initiation). After 30 min, [α-^32^P]CTP was added (pulse), the reaction was split into two equal volumes, and Zn^2+^ was added to a final concentration of 0.5 mM to one of the tubes. At the time points indicated, samples were taken and incorporation of [α-^32^P]CMP into viral RNA was analyzed. (**B**) Radiolabeled EAV RNA synthesized at the time points indicated above the lanes in the presence and absence of Zn^2+^. (**C**) Radiolabeled RNA synthesized by isolated SARS-CoV RTCs in reactions terminated after 100 (lane 1) and 40 (lane 2) min. Reaction products of a reaction to which 500 µM Zn^2+^ was added after 40 min, and that was terminated at t = 100 are shown in lane 3.

For EAV, close inspection of the RdRp assays revealed a less pronounced effect of Zn^2+^ on the generation of full-length 18-nt products than on the synthesis of smaller reaction intermediates (Fig. 5A). This suggested that Zn^2+^ specifically inhibited the initiation step of EAV RNA synthesis. To test this hypothesis, an RTC assay was incubated for 30 min with unlabeled CTP (initiation), after which the reaction was split in two. Then, [α-^32^P]CTP was added to both tubes (pulse), 500 µM Zn^2+^ was added to one of the tubes, and samples were taken at different time points during the reaction (Fig. 6A). Fig. 6B shows that in the presence of Zn^2+^ [α-^32^P]CMP was predominantly incorporated into nascent RNA molecules that were already past the initiation phase at the moment that Zn^2+^ was added to the reaction. No new initiation occurred, as was indicated by the smear of short radiolabeled products that progressively shifted up towards the position of full-length genomic RNA. This suggested that Zn^2+^ does not affect the elongation phase of EAV RNA synthesis and that it specifically inhibits initiation. This also explains the relatively weak signal intensity of the smaller sg mRNA bands (*e.g.*, compare the relative change in signal of RNA2 to RNA7) produced in the presence of Zn^2+^, since multiple initiation events are required on these short molecules to obtain signal intensities similar to those resulting from a single initiation event on the long genomic RNA, *e.g*., 16 times more in the case of RNA7. In contrast to EAV, the effect of Zn^2+^ on RNA synthesis by SARS-CoV RTCs was not limited to initiation, but appeared to impair the elongation phase as well, given that the addition of Zn^2+^ completely blocked further incorporation of [α-^32^P]CMP when added 40 min after the start of the reaction (Fig. 6C).

**Figure 7:**
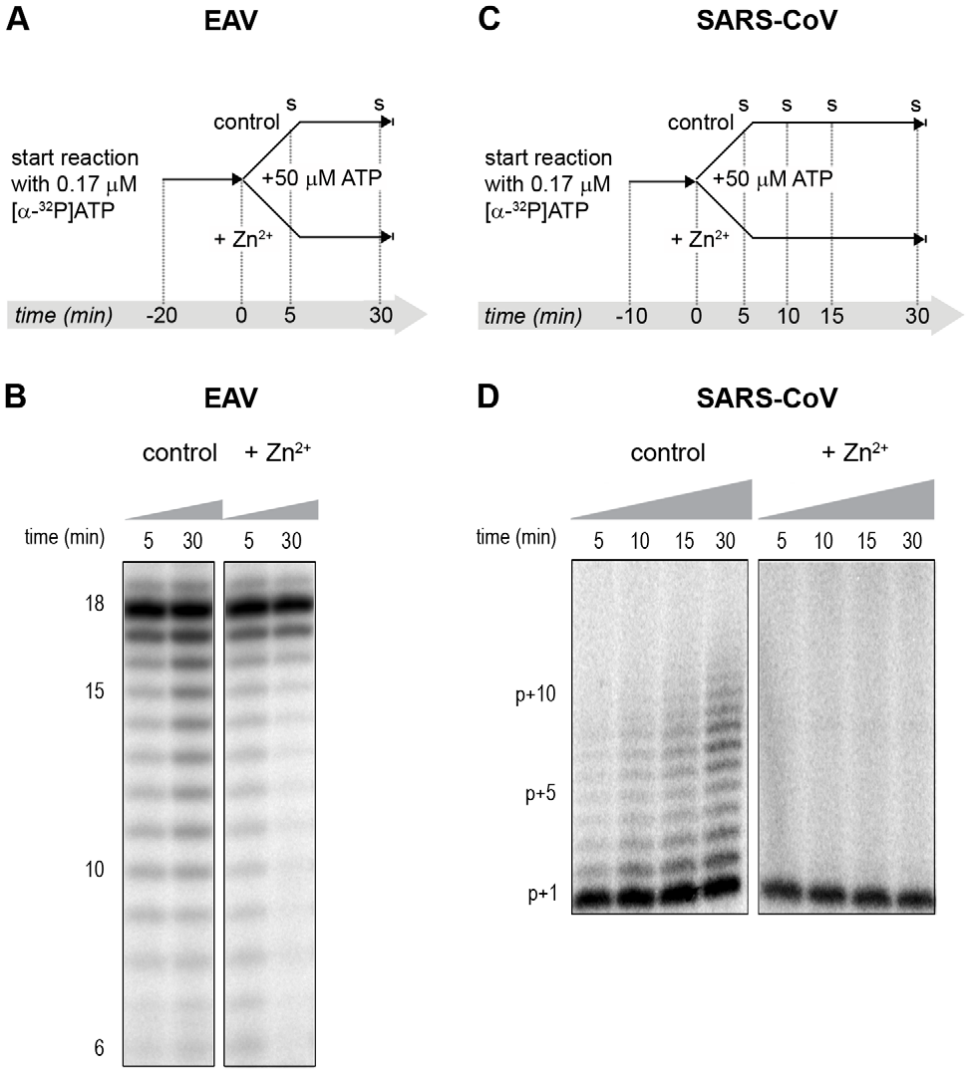
The effect of Zn^2+^ on initiation and elongation activity of purified EAV and SARS-CoV RdRps. (**A**) An EAV RdRp reaction was initiated in the presence of [α-^32^P]ATP under conditions that do not allow elongation, *i.e.*, low ATP concentration. After 20 min, the reaction was split into two equal volumes, and Zn^2+^ was added to one of the tubes. A chase with 50 µM unlabeled ATP, which allows elongation, was performed on both reactions and samples were taken after 5 and 30 min. (**B**) EAV RdRp reaction products that accumulated in the presence and absence of Zn^2+^ (indicated above the lanes) after a 5- and 30-min chase with unlabeled ATP. The length of the reaction products in nt is indicated next to the gel. (**C**) A SARS-CoV RdRp reaction was initiated in the presence of 0.17 µM [α-^32^P]ATP, which limits elongation. After 10 min, the reaction was split into two equal volumes, and Zn^2+^ was added to one of the tubes. A chase with 50 µM unlabeled ATP was performed on both reactions and samples were taken after 5, 10, 15, and 30 min. (**D**) SARS-CoV RdRp reaction products formed at the chase times indicated above the lanes in the presence and absence of Zn^2+^. The length of the reaction products in nt is indicated next to the gel (p is the primer length).

In the RdRp assays, the short templates used made it technically impossible to do experiments similar to those performed with complete RTCs. However, we previously noticed that at low concentrations of [α-^32^P]ATP (∼0.17 µM) SARS-CoV nsp12 RdRp activity was restricted to the addition of only a single nucleotide to the primer [27]. EAV nsp9 mainly produced very short (2–3 nt long) abortive RNA products and only a fraction of full length products, as is common for *de novo* initiating RdRps [28]. This allowed us to separately study the effect of Zn^2+^ on initiation and elongation by performing an experiment in which a pulse with a low concentration of [α-^32^P]ATP was followed by a chase in the presence of 50 µM of unlabeled ATP, which increased processivity and allowed us to study elongation (Fig. 7A and C) as described previously [27]. The results of these experiments were in agreement with those obtained with isolated RTCs. For EAV, with initiation and dinucleotide synthesis completely inhibited by the presence of 6 mM Zn^2+^ (Supplemental Fig. S2A), the amount of reaction intermediates shorter than 18 nt diminished with time, while products from templates on which the RdRp had already initiated were elongated to full-length 18-nt molecules (Fig. 7B, right panel). This was consistent with the observation that the EAV RdRp remained capable of extending the synthetic dinucleotide ApA to trinucleotides in the presence of Zn^2+^ (Supplemental Fig. S2B). Likely due to the absence of reinitiation in the reactions shown in Fig. 7B, the low processivity of the EAV RdRp, and the substrate competition between the remaining [α-^32^P]ATP and the >200 fold excess of unlabeled ATP, the differences between the 5- and 30-min time points were small. In the absence of Zn^2+^, the RdRp continued to initiate as indicated by the ladder of smaller-sized RNA molecules below the full-length product (Fig. 7B, left panel) and the time course shown in Supplemental Fig. S2A. In contrast, the addition of Zn^2+^ to a SARS-CoV RdRp reaction also blocked elongation, since extension of the radiolabeled primer as observed in the absence of Zn^2+^ (Fig. 7D, left panel) no longer occurred (Fig. 7D, right panel).

### Zinc affects SARS-CoV RdRp template binding

**Figure 8:**
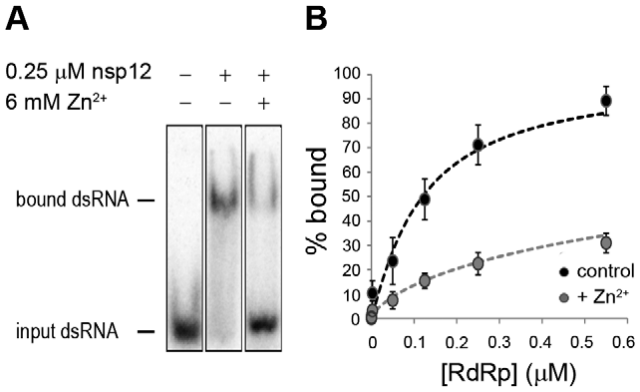
The effect of Zn^2+^ on SARS-CoV nsp12 template binding. (**A**) Electrophoretic mobility shift assay with radiolabeled dsRNA and nsp12 in the presence and absence of Zn^2+^ (indicated above the lanes). The position of unbound and nsp12-bound RNA in the gel is marked on the left of the panel. (**B**) Determination of the nsp12 affinity for RNA in the presence and absence of Zn^2+^. A fixed amount of RNA was incubated with an increasing amount of nsp12. This revealed a 3–4 fold reduction in the percentage of bound RNA in the presence of zinc ions (grey) relative to the percentage of bound RNA in the absence of zinc ions (black). Error bars represent standard error of the mean (n = 3).

To assess whether Zn^2+^ affects the interaction of recombinant SARS-CoV nsp12 with the template used in our assays, we performed electromobility shift assays (EMSA) in the presence and absence of Zn^2+^ (Fig. 8A). To measure the binding affinity of the RdRp for the template, we determined the fraction of bound template at various protein concentrations and observed a 3–4 fold reduction in RNA binding when Zn^2+^ was present in the assay (Fig. 8B). We also assessed whether pre-incubation of the RdRp or RNA with Zn^2+^ was a requirement for this drop in binding affinity, but found no significant difference with experiments not involving such a preincubation (data not shown).

No binding was observed when a similar RNA binding assay was performed with purified EAV RdRp. Likewise, nsp9 did not bind RNA in pull-down experiments with Talon-beads, His_6_-tagged nsp9, and radiolabeled EAV genomic RNA or various short RNA templates including polyU, whereas we were able to detect binding of a control protein (SARS-CoV nsp8, which has demonstrated RNA and DNA binding activity [32]) using this assay. It presently remains unclear why we are not able to detect the binding of recombinant EAV nsp9 to an RNA template.

## Discussion

Although a variety of compounds have been studied, registered antivirals are currently still lacking for the effective treatment of SARS and other nidovirus-related diseases [33]. RdRps are suitable targets for antiviral drug development as their activity is strictly virus-specific and may be blocked without severely affecting key cellular functions. Several inhibitors developed against the polymerases of e.g. human immunodeficiency virus (HIV) and hepatitis C virus are currently being used in antiviral therapy or clinical trials [34], [35], [36]. Therefore, advancing our molecular knowledge of nidovirus RdRps and the larger enzyme complexes that they are part of, and utilizing the potential of recently developed *in vitro* RdRp assays [25], [26], [27], [28] could ultimately aid in the development of effective antiviral strategies.

Zinc ions and zinc-ionophores, such as PT and PDTC, have previously been described as potent inhibitors of various RNA viruses. We therefore investigated whether PT-stimulated import of zinc ions into cells also inhibited the replication of nidoviruses in cell culture. Using GFP-expressing EAV and SARS-CoV [29], [30], we found that the combination of 2 µM PT and 2 µM Zn^2+^ efficiently inhibited their replication, while not causing detectable cytoxicity (Fig. 1). Inhibition of replication by PT and Zn^2+^ at similar concentrations (2–10 µM) was previously observed for several picornaviruses such as rhinoviruses, foot-and-mouth disease virus, coxsackievirus, and mengovirus [6], [7], [8], [9], [10], [11].

The inhibitory effect of Zn^2+^ on the replication of picornaviruses appeared to be due to interference with viral polyprotein processing. In infections with the coronavirus mouse hepatitis virus (MHV), Zn^2+^ also interfered with some of the replicase polyproteins cleavages [24], albeit at a much higher concentration (100 µM Zn^2+^) than used in our studies. Since impaired replicase processing will indirectly affect viral RNA synthesis in the infected cell, we used two recently developed *in vitro* assays to investigate whether Zn^2+^ also affects nidovirus RNA synthesis directly. Our *in vitro* studies revealed a strong inhibitory effect of zinc ions on the RNA-synthesizing activity of isolated EAV and SARS-CoV RTCs. Assays with recombinant enzymes subsequently demonstrated that this was likely due to direct inhibition of RdRp function. The inhibitory effect could be reversed by chelating the zinc ions, which provides an interesting experimental (on/off) approach to study nidovirus RNA synthesis. Addition of Zn^2+^ following initiation of EAV RNA synthesis had little or no effect on NTP incorporation in molecules whose synthesis had already been initiated in the absence of Zn^2+^ (Fig. 6 and 7), indicating that Zn^2+^ does not affect elongation and does not increase the termination frequency, as was previously found for Mn^2+^ [25]. Therefore, Zn^2+^ appears to be a specific inhibitor of the initiation phase of EAV RNA synthesis. In contrast, Zn^2+^ inhibited SARS-CoV RdRp activity also during the elongation phase of RNA synthesis, probably by directly affecting template binding (Fig. 8). In coronaviruses, zinc ions thus appear to inhibit both the proper proteolytic processing of replicase polyproteins [23], [24] and RdRp activity (this study). Contrary to the RTC assays, millimolar instead of micromolar concentrations of ZnOAc_2_ were required for a nearly complete inhibition of nucleotide incorporation in RdRp assays.

It has been well established that DNA and RNA polymerases use a triad of conserved aspartate residues in motifs A and C to bind divalent metal ions like Mg^2+^, which subsequently coordinate incoming nucleotides during the polymerization reaction [37], [38]. Mg^2+^ is also the divalent metal ion that is required for the *in vitro* activity of isolated SARS-CoV and EAV RTCs and recombinant RdRps [25], [26], [27], [28], although *de novo* initiation of EAV nsp9 is primarily Mn^2+^-dependent. Zn^2+^ could not substitute for Mg^2+^ or Mn^2+^ as cofactor as it was incapable of supporting the polymerase activity of nidovirus RTCs and RdRps in the absence of Mg^2+^ (data not shown), as was also reported for the poliovirus RdRp [39]. Moreover, inhibition of nidovirus RdRp activity by Zn^2+^ was even observed at low concentrations and in the presence of a more than 25-fold excess of Mg^2+^, suggesting that either the affinity of the active site for Zn^2+^ is much higher or that Zn^2+^ does not compete for Mg^2+^-binding and binds to another zinc(-specific) binding site in the protein.

Specific protein domains or pockets that contain zinc ions may be involved in protein-protein interactions, protein-RNA/DNA interactions, or conformational changes in enzyme structures. Zinc-binding domains commonly consist of at least three conserved cysteine and/or histidine residues within a stretch of ∼10–30 amino acids, such as in zinc-finger motifs and metalloproteases [2], [40], [41]. However, in RdRps there are only few precedents for the presence of zinc-binding pockets, such as those identified in the crystal structure of the Dengue RdRp [42]. Sequence analysis of the EAV nsp9 amino acid sequence revealed that it lacks patches rich in conserved cysteines and/or histidines. In contrast, inspection of the SARS-CoV nsp12 amino acid sequence revealed two such patches, namely H295-C301-C306-H309-C310 and C799-H810-C813-H816. A crystal structure for nsp12 is presently unavailable, but a predicted structure that represents the C-terminal two-thirds of the enzyme has been published [31]. Interestingly, in this model, C799, H810, C813 and H816 are in a spatial arrangement resembling that of the Zn^2+^ coordinating residues in the Zn2 zinc-binding pocket found in motif E of the Dengue virus RdRp (see Supplemental Fig. S3). Clearly, an in-depth analysis of nidovirus RdRps, e.g. through structural analysis and subsequent mutational studies targeting aforementioned cysteines and histidines, is required to provide further insight into and a structural basis for the Zn^2+^-induced inhibitory effects on RdRp activity documented in this study. Such studies may, however, be complicated when Zn^2+^ binding proves to be very transient in nature and not detectable with currently available methods.

In summary, the combination of zinc ions and the zinc-ionophore PT efficiently inhibits nidovirus replication in cell culture. This provides an interesting basis for further studies into the use of zinc-ionophores as antiviral compounds, although systemic effects have to be considered [43], [44] and a water-soluble zinc-ionophore may be better suited, given the apparent lack of systemic toxicity of such a compound at concentrations that were effective against tumors in a mouse xenograft model [45]. *In vitro*, the reversible inhibition of the RdRp by Zn^2+^ has also provided us with a convenient research tool to gain more insight into the molecular details of (nido)viral RNA synthesis, and revealed novel mechanistic differences between the RdRps of SARS-CoV and EAV.

## Materials and Methods

### Cells and viruses

Vero-E6 cells were cultured and infected with SARS-CoV (strain Frankfurt-1; accession nr. AY291315) or SARS-CoV-GFP as described previously [46]. All procedures involving live SARS-CoV were performed in the biosafety level 3 facility at Leiden University Medical Center. BHK-21 or Vero-E6 cells were cultured and infected with EAV (Bucyrus strain; accession nr. NC_002532) or EAV-GFP [29] as described elsewhere [25].

### Effect of zinc ions on nidovirus replication in cell culture

One day prior to infection, Vero-E6 cells were seeded in transparent or black (low fluorescence) 96-well clusters at 10,000 cells per well. The next day, cells were infected with SARS-CoV-GFP or EAV-GFP with an m.o.i. of 4, and 1 h p.i. the inoculum was removed and 100 µl of medium containing 2% fetal calf serum (FCS) was added to each well. In some experiments 0–32 µM of pyrithione (Sigma) was added in addition to 0–2 µM ZnOAc_2_. Infected cells were fixed at 17 h p.i. by aspirating the medium and adding 3% paraformaldehyde in PBS. After washing with PBS, GFP expression was quantified by measuring fluorescence with a LB940 Mithras plate reader (Berthold) at 485 nm. To determine toxicity of ZnOAc_2_ and PT, cells were exposed to 0–32 µM PT and 0–8 µM ZnOAc_2_. After 18 h incubation, cell viability was determined with the Cell Titer 96 AQ MTS assay (Promega). EC_50_ and CC_50_ values were calculated with Graphpad Prism 5 using the nonlinear regression model.

### RNA templates and oligonucleotides

RNA oligonucleotides SAV557R (5′-GCUAUGUGAGAUUAAGUUAU-3′), SAV481R (5′-UUUUUUUUUUAUAACUUAAUCUCACAUAGC-3′) and poly(U)_18_ (5′-UUUUUUUUUUUUUUUUUU-3′) were purchased from Eurogentec, purified from 7 M Urea/15% PAGE gels and desalted through NAP-10 columns (GE healthcare). To anneal the RNA duplex SAV557R/SAV481R, oligonucleotides were mixed at equimolar ratios in annealing buffer (20 mM Tris-HCl pH 8.0, 50 mM NaCl and 5 mM EDTA), denatured by heating to 90°C and allowed to slowly cool to room temperature after which they were purified from 15% non-denaturing PAGE gels.

### *In vitro* viral RNA synthesis assay with isolated RTCs

SARS-CoV and EAV RTCs were isolated from infected cells and assayed for activity *in vitro* as described previously [25], [26]. To assess the effect of Zn^2+^, 1 µl of a ZnOAc_2_ stock solution was added to standard 28-µl reactions, resulting in final Zn^2+^ concentrations of 10–500 µM. When Zn^2+^ had to be chelated in the course of the reaction, magnesium-saturated EDTA (MgEDTA) was added to a final concentration of 1 mM. After RNA isolation, the ^32^P-labeled reaction products were separated on denaturing 1% (SARS-CoV) or 1.5% (EAV) agarose formaldehyde gels. The incorporation of [α-^32^P]CMP into viral RNA was quantified by phosphorimaging of the dried gels using a Typhoon scanner (GE Healthcare) and the ImageQuant TL 7 software (GE Healthcare).

### Expression and purification of nidovirus RdRps

SARS-CoV nsp12 and EAV nsp9 were purified essentially as described elsewhere [27], [28], but with modifications for nsp9. In short, *E. coli* BL21(DE3) with plasmid pDEST14-nsp9-CH was grown in auto-induction medium ZYM-5052 [47] for 6 hours at 37°C and a further 16 hours at 20°C. After lysis in buffer A (20 mM HEPES pH 7.4, 200 mM NaCl, 20 mM imidazole, and 0.05% Tween-20) the supernatant was applied to a HisTrap column (GE Healthcare). Elution was performed with a gradient of 20–250 mM imidazole in buffer A. The nsp9-containing fraction was further purified by gel filtration in 20 mM HEPES, 300 mM NaCl and 0.1% Tween-20 on a Superdex 200 column (GE Healthcare). The fractions containing nsp9-CH were pooled, dialyzed against 1000 volumes of buffer B (20 mM HEPES, 100 mM NaCl, 1 mM DTT and 50% glycerol) and stored at −20°C. RdRps with a D618A (SARS-CoV) or D445A (EAV) mutation were obtained by site-directed mutagenesis of the wild-type (wt) plasmid pDEST14-nsp9-CH [28] with oligonucleotides 5′-TACTGCCTTGAAACAGCCCTGGAGAGTTGTGAT-3′ and 5′-ATCACAACTCTCCAGGGCTGTTTCAAGGCAGTA-3′, and plasmid pASK3-Ub-nsp12-CHis_6_ with oligonucleotides 5′-CCTTATGGGTTGGGCTTATCCAAAATGTG-3′ and 5′-CACATTTTGGATAAGCCCAACCCATAAGGA-3′, as described elsewhere [27]. Mutant proteins were purified parallel to the wt enzymes.

### RdRp assays with purified enzymes

Standard reaction conditions for the RdRp assay with 0.1 µM of purified SARS-CoV nsp12 are described elsewhere [27]. To study the effect of Zn^2+^ in this assay, 0.5 µl of a dilution series of 0–80 mM ZnOAc_2_ was added to the 5 µl reaction mixture, yielding final Zn^2+^ concentrations of 0–8 mM. The EAV RdRp assay contained 1 µM nsp9, 1 µM RNA template poly(U)_18_, 0.17 µM [α-^32^P]ATP (0.5 µCi/µl; Perkin-Elmer), 50 µM ATP, 20 mM Tris-HCl (pH 8.0), 10 mM NaCl, 10 mM KCl, 1 mM MnCl_2_, 4 mM MgOAc_2_, 5% glycerol, 0.1% Triton-X100, 1 mM DTT and 0.5 units RNaseOUT. ZnOAc_2_ was added to the reaction to give a final concentration of 0–6 mM. To chelate Zn^2+^ during reactions, MgEDTA was added to a final concentration of 8 mM. Reactions were terminated after 1 hour and analyzed as described [27].

### SARS-CoV nsp12 electrophoretic mobility shift assay

SARS-CoV RdRp was incubated with 0.2 nM 5′ ^32^P-labeled SAV557R/SAV481R RNA duplex, for 10 minutes at 30°C either in presence or absence of 6 mM ZnOAc_2_. Reactions were analyzed as described previously [27].

## Supporting Information

Figure S1 **Effect of various divalent cations on the RdRp activity of SARS-CoV nsp12.** Purified recombinant SARS-CoV nsp12 was incubated with a primed template, ATP, and [α-^32^P]ATP in the presence of either 6 mM Mg^2+^ only (lane 1), and with increasing concentrations of a second divalent metal (M^2+^), specifically: 2–6 mM Ca^2+^ (lane 2–4), 2–6 mM Co^2+^ (lane 5–7), 2–6 mM Zn^2+^ (lane 8–10), or 2–6 mM Mn^2+^ (lane 11–13). The strongest inhibition was observed for Zn^2+^. For more details on the SARS-CoV nsp12 RdRp assay, see the main text.
(1.55 MB TIF)

Figure S2 **Effect of Zn^2+^ on the dinucleotide extension activity of EAV nsp9.** Purified recombinant EAV nsp9 was incubated with a U^18^ template in the presence of [α-^32^P]ATP, ATP, 4 mM Mg^2+^, 1 mM Mn^2+^, and 1 µM ApA. (**A**) Reaction mixtures were split into two aliquots, one of which was supplemented with 6 mM Zn^2+^, and samples were taken at the time points (minutes) indicated above the lanes. In the absence of Zn^2+^, EAV nsp9 initiates *de novo* and produces di- and trinucleotides, indicated with A2 and A3, respectively. A non-specific band, unrelated to RdRp activity, between A2 and A3 is indicated with an asterisk. In the presence of 6 mM Zn^2+^, the synthesis of dinucleotides and trinucleotides was blocked. (**B**) When performing the assay described under (A) in the absence of Zn^2+^, a full-length product of 18 nucleotides is formed. This product is not observed when the assay is performed in the presence of 6 mM Zn^2+^, but nsp9 was capable of elongating the provided dinucleotide primer ApA into tri- (ApA*pA) and tetranucleotide ((ApA*pA*pA) products (the asterisk indicates radiolabeled phosphates). Due to the absence of a 5′ triphosphate group, these reaction products migrate much slower in the 20% acrylamide and 7 M urea gel used for this analysis. See the main text for additional experimental details on the EAV nsp9 RdRp assay.
(2.16 MB TIF)

Figure S3 **Putative zinc-binding residues in the predicted structure of SARS-CoV nsp12 and comparison with the structure of the zinc-containing Dengue virus RdRp domain.** (**A**) Sequence alignment of coronavirus RdRps showing conservation of four potential zinc-binding residues amino acids (C799-H810-C813-H816 in SARS-CoV; indicated with asterisks) in the C-terminal region of coronavirus nsp12. Black shading indicates complete conservation among coronaviruses. The coronavirus RdRp sequences were aligned with Muscle 3.6. The aligned sequences and NCBI accession numbers are the following: mouse hepatitis virus strain A59 (MHV_A59; NP_068668), human CoV 229E (HCoV_229E; NP_068668), infectious bronchitis virus strain Beaudette (IBV_B; P0C6Y1), bovine coronavirus (BCoV; NP_742138.1), feline coronavirus (FeCoV; YP_239353.1), and SARS-CoV strain Frankfurt-1 (SARS_Fr1; AAP33696). (**B**) Crystal structure of the Dengue virus RdRp domain showing the position of four cysteine and histidine residues that form Zn^2+^-binding pocket Zn2, located close to motif E (depicted in red). A second Zn^2+^-binding pocket (Zn1) and the two zinc ions identified in the crystal structure are indicated in blue-gray. (**C**) Predicted three-dimensional structure model of SARS-CoV nsp12 (Xu et al., Nucl. Acids Res. 31: 7117–7130), based on PDB code 1O5S, rendered with Swiss-PdbViewer 4.01 and POV-Ray 3.6. The positions of the conserved cysteine and histidine residues indicated in panel A (C799-H810-C813-H816) close to motif E (depicted in red) and RdRp active-site residues (D618, D760 and D761) are indicated. The spatial arrangement of these cysteines and histidines in this model strikingly resembles the positioning of the metal ion-coordinating residues of Zn-binding pocket Zn2 in the Dengue virus RdRp domain (see panel B).
(0.86 MB TIF)
