# Supplementary figures and images for: Zn2+ Inhibits Coronavirus and Arterivirus RNA Polymerase Activity In Vitro and Zinc Ionophores Block the Replication of These Viruses in Cell Culture
Source: PLoS Pathog. 2010 Nov 4;6(11):e1001176. doi: 10.1371/journal.ppat.1001176 (PMC2973827; doi:10.1371/journal.ppat.1001176)

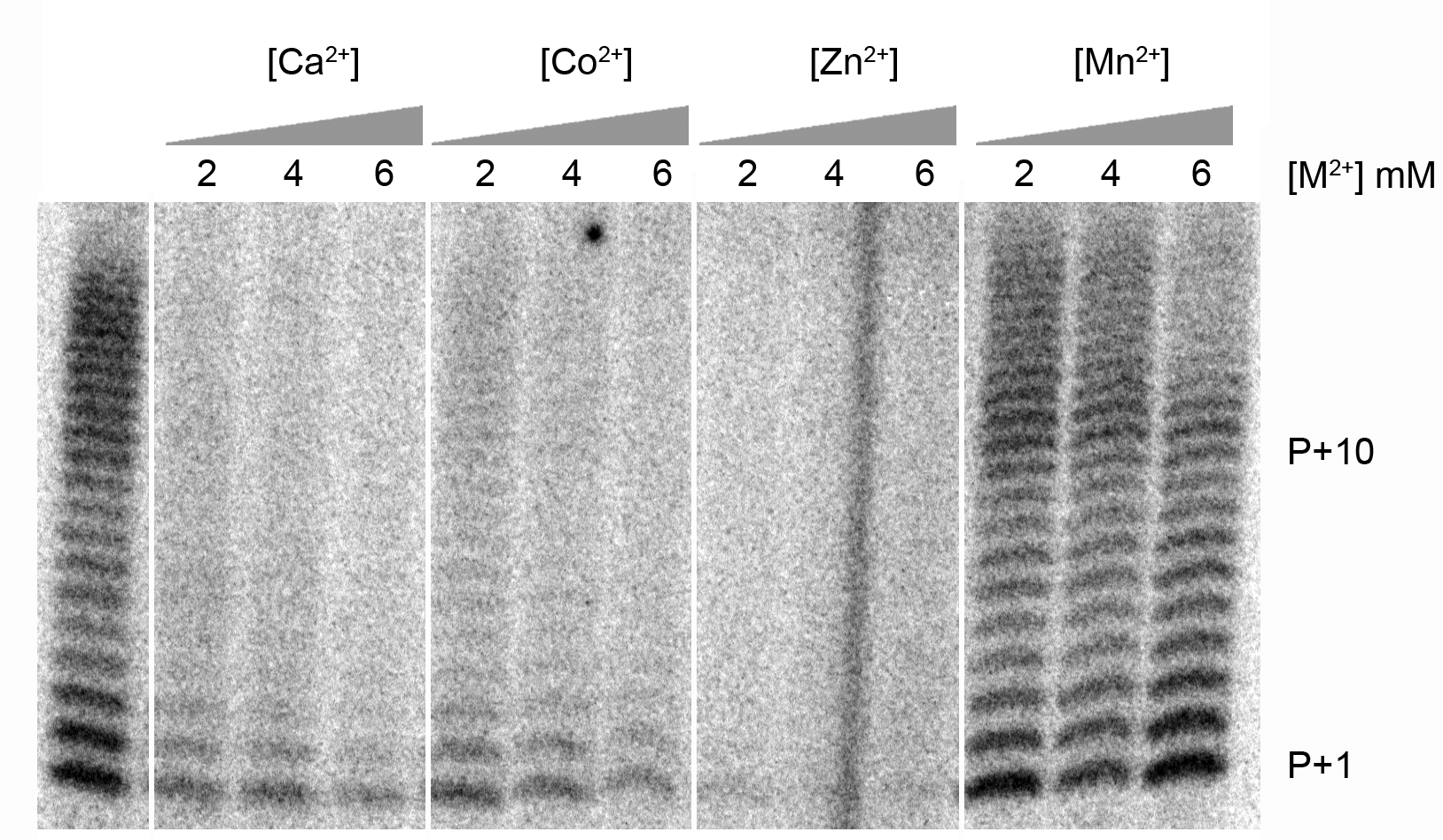

Supplement: Figure S1 — Effect of various divalent cations on the RdRp activity of SARS-CoV nsp12. Purified recombinant SARS-CoV nsp12 was incubated with a primed template, ATP, and [α-32P]ATP in the presence of either 6 mM Mg2+ only (lane 1), and with increasing concentrations of a second divalent metal (M2+), specifically: 2–6 mM Ca2+ (lane 2–4), 2–6 mM Co2+ (lane 5–7), 2–6 mM Zn2+ (lane 8–10), or 2–6 mM Mn2+ (lane 11–13). The strongest inhibition was observed for Zn2+. For more details on the SARS-CoV nsp12 RdRp assay, see the main text. (1.55 MB TIF) [file ppat.1001176.s001.tif]

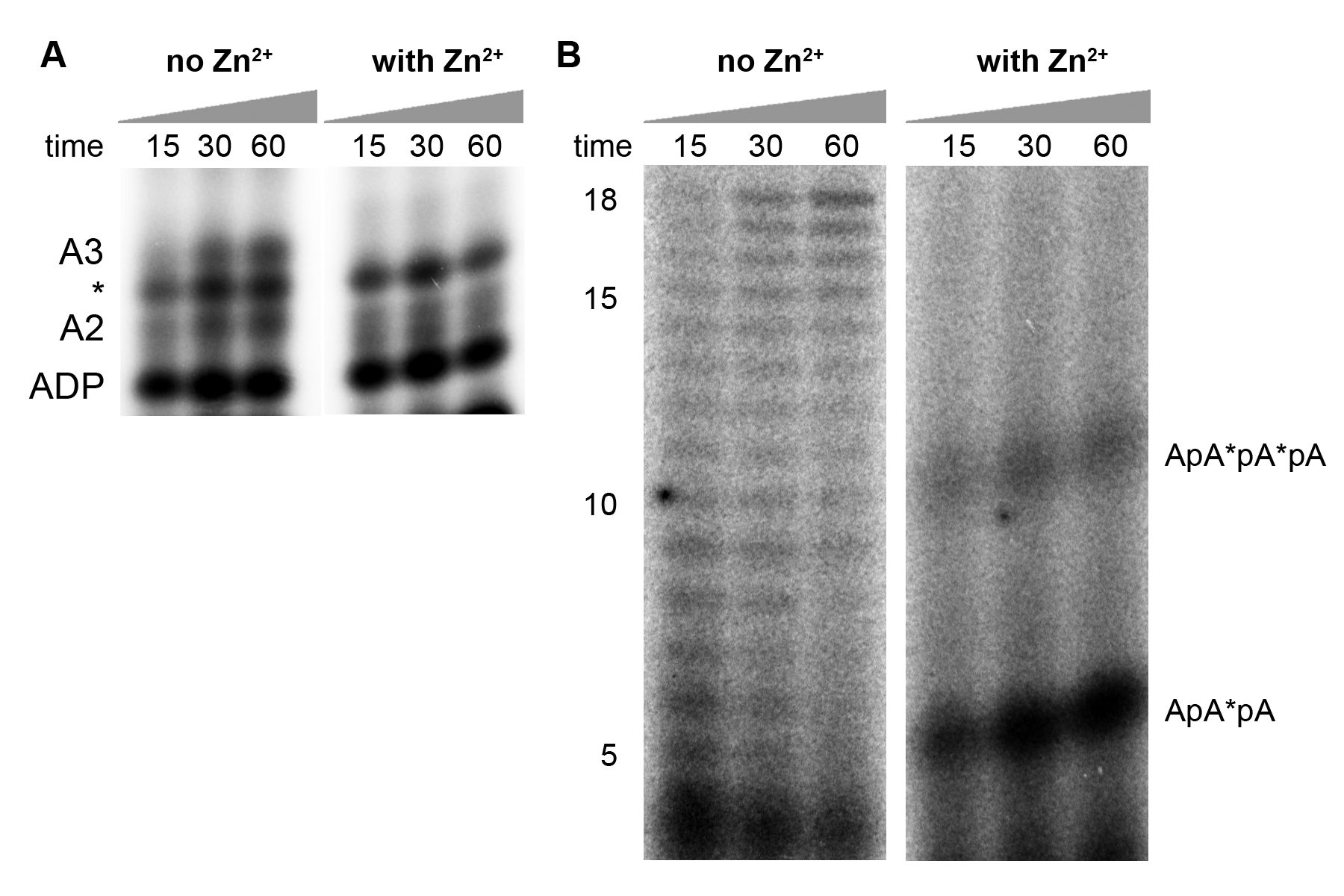

Supplement: Figure S2 — Effect of Zn2+ on the dinucleotide extension activity of EAV nsp9. Purified recombinant EAV nsp9 was incubated with a U18 template in the presence of [α-32P]ATP, ATP, 4 mM Mg2+, 1 mM Mn2+, and 1 µM ApA. (A) Reaction mixtures were split into two aliquots, one of which was supplemented with 6 mM Zn2+, and samples were taken at the time points (minutes) indicated above the lanes. In the absence of Zn2+, EAV nsp9 initiates de novo and produces di- and trinucleotides, indicated with A2 and A3, respectively. A non-specific band, unrelated to RdRp activity, between A2 and A3 is indicated with an asterisk. In the presence of 6 mM Zn2+, the synthesis of dinucleotides and trinucleotides was blocked. (B) When performing the assay described under (A) in the absence of Zn2+, a full-length product of 18 nucleotides is formed. This product is not observed when the assay is performed in the presence of 6 mM Zn2+, but nsp9 was capable of elongating the provided dinucleotide primer ApA into tri- (ApA*pA) and tetranucleotide ((ApA*pA*pA) products (the asterisk indicates radiolabeled phosphates). Due to the absence of a 5′ triphosphate group, these reaction products migrate much slower in the 20% acrylamide and 7 M urea gel used for this analysis. See the main text for additional experimental details on the EAV nsp9 RdRp assay. (2.16 MB TIF) [file ppat.1001176.s002.tif]

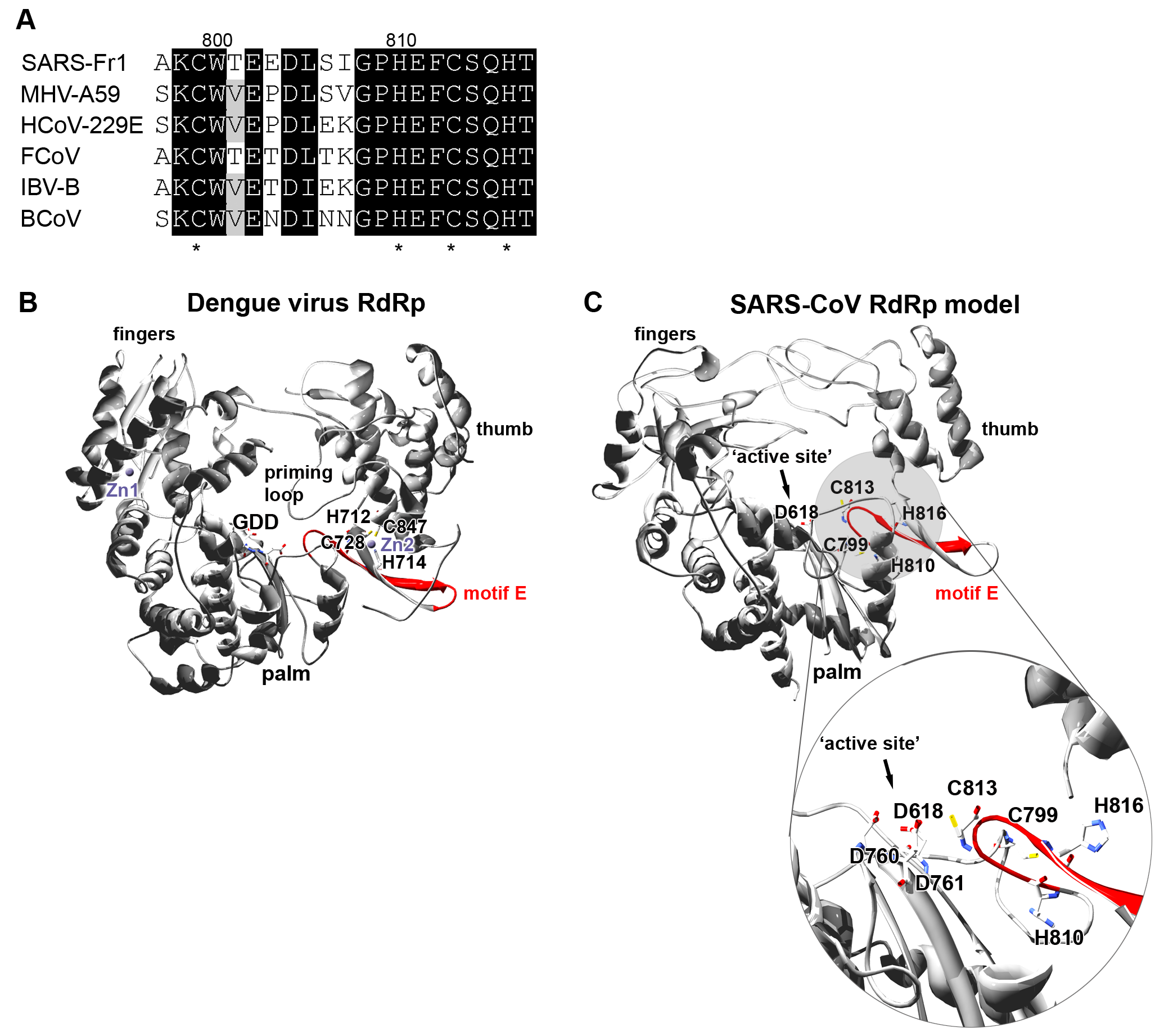

Supplement: Figure S3 — Putative zinc-binding residues in the predicted structure of SARS-CoV nsp12 and comparison with the structure of the zinc-containing Dengue virus RdRp domain. (A) Sequence alignment of coronavirus RdRps showing conservation of four potential zinc-binding residues amino acids (C799-H810-C813-H816 in SARS-CoV; indicated with asterisks) in the C-terminal region of coronavirus nsp12. Black shading indicates complete conservation among coronaviruses. The coronavirus RdRp sequences were aligned with Muscle 3.6. The aligned sequences and NCBI accession numbers are the following: mouse hepatitis virus strain A59 (MHV_A59; NP_068668), human CoV 229E (HCoV_229E; NP_068668), infectious bronchitis virus strain Beaudette (IBV_B; P0C6Y1), bovine coronavirus (BCoV; NP_742138.1), feline coronavirus (FeCoV; YP_239353.1), and SARS-CoV strain Frankfurt-1 (SARS_Fr1; AAP33696). (B) Crystal structure of the Dengue virus RdRp domain showing the position of four cysteine and histidine residues that form Zn2+-binding pocket Zn2, located close to motif E (depicted in red). A second Zn2+-binding pocket (Zn1) and the two zinc ions identified in the crystal structure are indicated in blue-gray. (C) Predicted three-dimensional structure model of SARS-CoV nsp12 (Xu et al., Nucl. Acids Res. 31: 7117–7130), based on PDB code 1O5S, rendered with Swiss-PdbViewer 4.01 and POV-Ray 3.6. The positions of the conserved cysteine and histidine residues indicated in panel A (C799-H810-C813-H816) close to motif E (depicted in red) and RdRp active-site residues (D618, D760 and D761) are indicated. The spatial arrangement of these cysteines and histidines in this model strikingly resembles the positioning of the metal ion-coordinating residues of Zn-binding pocket Zn2 in the Dengue virus RdRp domain (see panel B). (0.86 MB TIF) [file ppat.1001176.s003.tif]
